# Advancing Our Understanding of the Link between Statistical Learning and Language Acquisition: The Need for Longitudinal Data

**DOI:** 10.3389/fpsyg.2012.00324

**Published:** 2012-08-31

**Authors:** Joanne Arciuli, Janne von Koss Torkildsen

**Affiliations:** ^1^Faculty of Health Sciences, University of SydneySydney, NSW, Australia; ^2^Department of Biological and Medical Psychology, University of BergenBergen, Norway

**Keywords:** statistical learning, language acquisition, longitudinal studies, language impairment, language proficiency

## Abstract

Mastery of language can be a struggle for some children. Amongst those that succeed in achieving this feat there is variability in proficiency. Cognitive scientists remain intrigued by this variation. A now substantial body of research suggests that language acquisition is underpinned by a child’s capacity for statistical learning (SL). Moreover, a growing body of research has demonstrated that variability in SL is associated with variability in language proficiency. Yet, there is a striking lack of longitudinal data. To date, there has been no comprehensive investigation of whether a capacity for SL in young children is, in fact, associated with language proficiency in subsequent years. Here we review key studies that have led to the need for this longitudinal research. Advancing the language acquisition debate via longitudinal research has the potential to transform our understanding of typical development as well as disorders such as autism, specific language impairment, and dyslexia.

Statistical learning (SL) likely plays a role in a large number of perceptual and cognitive activities. For example, “Every time we listen to a blues song or a piano concerto, our brains pick up on the underlying statistics regarding which notes tend to occur together or follow one another in these different styles. We use this accumulated knowledge to appraise unfamiliar pieces of music or different performances of well-known songs. In short, our expectations are an outcome of statistical learning” (Janata, 2006, p. 29). The role of SL during language acquisition has been hotly debated over several decades. Certainly, it is clear that language contains many statistical regularities. It has been suggested that SL operates on these regularities and facilitates processes as varied as word segmentation, vocabulary learning, and syntax (Rowland and Pine, 2000; Finn and Hudson Kam, 2008; Yu, 2008).

Consider word segmentation. Child-directed speech includes utterances such as *prettydolly* and *prettykitty*. Each utterance is composed of two words, usually spoken as a continuous stream without pausing between words. How do children identify the separate words (*pretty*, *dolly*, and *kitty*)? Perhaps they detect implicitly the strength of associations between adjacent syllables; *pre* is often followed by *tty* (high joint probability), however, *tty* is rarely followed by *do* (low joint probability). In natural language, joint probabilities between syllables are highest within words; those spanning word boundaries are lower. Thus, sensitivity to these co-occurrence statistics might assist children to segment the speech stream into words, possibly in conjunction with other cues such as prosody (Hay and Saffran, in press). Newman et al. (2006) discovered a relationship between infants’ ability to segment the speech stream into words and language proficiency at 24 months and, later, between 4 and 6 years. It was shown that IQ did not mediate this relationship.

As elegant as this theory of language acquisition is, there remain striking gaps in our understanding of the link between SL and language. To date, there has been no direct investigation of whether a capacity for SL in young children is, in fact, associated with language proficiency in subsequent years. This paper provides a brief introduction to the language acquisition debate, a review of key studies indicating a link between SL ability and language proficiency, and a discussion of the kind of longitudinal research that is needed in order to advance the debate about the role of learning during language acquisition. We argue that this kind of longitudinal research will enhance our understanding of language development in typically developing children and, potentially, transform our understanding of disorders such as autism, specific language impairment (SLI), and dyslexia.

## Debate about the Nature of Language Acquisition

There has been a long-standing debate about the link between learning and language acquisition. Chomsky and others have speculated that language is too complex and the learning environment too impoverished to be assisted by a general learning mechanism (e.g., Chomsky, 1975; Pinker, 1989; Crain, 1991; special issue edited by Ritter, 2002). This led to the suggestion that children come into the world already equipped with a great deal of linguistic knowledge. The innateness hypothesis incorporates multiple and intertwined notions including both linguistic universals and modularity. While these cannot be covered adequately here, Evans and Levinson (2009) and Hulme and Snowling (2009) provide contemporary discussion of linguistic universals and of modularity in relation to children’s development, respectively.

The language acquisition debate has been reinvigorated by the emergence of large language databases in combination with powerful computing resources which have revealed surprisingly rich statistical structure in natural language. Moreover, a now substantial body of research indicates that, from a very young age, the brain can detect these statistical regularities. This appears to occur even under challenging learning conditions (e.g., when only positive evidence is available; when stimuli are presented briefly; when there are irregularities in the input). Key studies from these bodies of research are reviewed in subsequent sections of this paper.

For some, the debate does not center on a distinction between innateness versus learning, but rather on the relative contributions of these (Gould and Marler, 1987; Yang, 2004; Gervain and Mehler, 2010). Yang (2006) suggested that “A somewhat curious response to the mystery of grammar learning is to say that there is basically *no* learning…for the unfortunate few who do experience language learning problems, getting a detailed understanding of how language learning takes place is probably well worthwhile” (pp. 150–152). An unresolved question is whether some aspects of language, such as grammatical structure, are less learnable and more heavily underpinned by innate knowledge than others (e.g., Nowak et al., 2002; Peña et al., 2002; Seidenberg et al., 2002). Bayesian models have representational flexibility allowing a move away from some conventional dichotomies that have shaped language acquisition research. For example, Perfors et al. (2011) explored the learning of phrase structure in the context of typical child-directed speech and innate domain-general capacities. Others have suggested that there is a shift from general learning mechanisms to language specific processes across development (Namy, 2012).

## Statistical Learning

Statistical learning has been described as “automatic,” “incidental,” and “spontaneous.” Perruchet and Pacton (2006) argued that SL is a form of implicit learning in that participants in SL experiments are presented with structured material and are not given any instructed regarding learning; they learn from exposure to positive instances.

Statistical learning of regularities can be assessed in a number of ways. One method is the long-established sequential learning paradigm which utilizes embedded triplets to determine sensitivity to adjacent dependencies. The paradigm can be used with either auditory or visual stimuli. For instance, a child may be asked to watch a continuous sequence of evenly paced individually presented items (represented here by letters). Typically, each item appears for around 400 ms. The sequence contains embedded triplets such as A–P–K; for example, … L–A–P–K–G–H–D–A–P–K–X …. After several minutes of watching this *familiarization stream*, the experimenter surprises the child with *test phase*: during forced-choice trials two triplets are presented in succession (the three component items of the triplet are displayed individually, one triplet then the other). One of these triplets, the embedded triplet, had been repeated during the continuous sequence while the other, a foil, had never appeared. The child judges which of the triplets is familiar (e.g., APK or AXG?). Most identify the embedded triplets as familiar, even though there was no advance warning of patterns and no reinforcement. Generally, participants have no conscious sense of familiarity. Data are analyzed to determine whether performance is significantly different from chance (using a one-sample *t*-test comparing the group average for percentage of correctly identified embedded triplets against chance, which is 50%).

Studies focusing on infants have utilized this paradigm or similar ones, but require a different kind of responding during the test phase (e.g., headturn preference). A seminal study in *Science* revealed that 8-month-olds can learn the strength of sequential associations between syllables in pseudospeech after only 2 min of exposure (Saffran et al., 1996). Recently, another study demonstrated that 8-month-olds are able to track such transitional probabilities also in *natural language* (Pelucchi et al., 2009). A study using sequences of visually presented shapes showed SL in 2-month-olds (Kirkham et al., 2002).

Many studies of SL have examined the ability to detect associations among adjacent items that are presented sequentially; however, natural language also contains *non-adjacent patterns* (e.g., syntactic structure can involve dependencies among elements that are distant from one another). SL can also operate on non-adjacent patterns (Newport and Aslin, 2004). Recent studies using the event-related potential (ERP) technique have found that the ability to extract statistical dependencies between adjacent elements in the speech stream appears to be present from birth, and that infants can learn non-adjacent dependencies in a natural, non-native language by 4 months of age (Teinonen et al., 2009; Friederici et al., 2011). Some aspects of language processing may require *spatial* rather than sequential learning (e.g., certain aspects of orthography; aspects of sign language). SL has been shown to operate on spatial regularities (e.g., Fiser and Aslin, 2005).

Statistical learning does not decay rapidly. Kim et al. (2009) exposed participants to statistical regularities present in a familiarization stream 24 h before the test phase and showed significant learning despite the delay. Arciuli and Simpson (2012a) replicated this finding. In addition, they demonstrated that SL is remarkably consistent regardless of whether familiarization and test phase are separated by 30 min, 1, 2, 4, or 24 h. Participants still showed significant learning. Neuroscientific evidence has confirmed that SL operates without instruction to learn, and in those who had no conscious sense of familiarity during the test phase (Turk-Browne et al., 2009).

Many SL paradigms, such as the triplet paradigm described above, measure participants’ recognition of the exact stimuli that was used during familiarization. SL studies have also included measures of generalization; that is, whether participants can learn regularities from one set or stimuli and subsequently apply their implicit knowledge to stimuli they have never encountered before (Gómez and Gerken, 2000). Generalization indicates that learners have moved beyond recognition of specific items to an understanding of the underlying patterns they represent. Studies of infants have shown that the ability to generalize regularities in language is already present in the first year of life (Marcus et al., 1999; Gerken and Bollt, 2008) and is so robust that infants can generalize a predominant grammatical pattern even when they are faced with inconsistent input (Gómez and Lakusta, 2004).

Gómez and Lakusta found that 12-month-olds were able to abstract form-based categories in an artificial grammar where only 83% of the training strings represented the correct grammar and the remaining 17% represented a different structure which was inconsistent with the grammar. Furthermore, results from studies using generalization paradigms indicate that variability (e.g., in terms of the number of different exemplars representing a structure) is a key factor which facilitates generalization (Wonnacott et al., 2012). It is still unclear, however, whether this finding carries over to complex natural language settings (van Heugten and Johnson, 2010).

The role of sleep is particularly interesting with regard to the difference between SL paradigms that test recognition and SL paradigms that test generalization. Arciuli and Simpson (2012a) found that adults’ recognition of the exact stimuli presented during familiarization was not affected by sleep. This result is consistent with a study by Nemeth et al. (2010) showing no effect of sleep on subjects’ ability to learn specific motor sequences in an implicit learning task. However, a different picture emerges from the studies that have investigated subjects’ ability to generalize their learning to novel cases. Gómez et al. (2006) compared non-adjacent dependency learning in infants who napped between familiarization and testing to infants who did not sleep. Results showed that the no-nap group preferred listening to familiar over unfamiliar trials, consistent with veridical memory of specific non-adjacent phrases. Infants in the nap group, however, listened longer to sentences conforming to the grammar, but did not distinguish between familiar and unfamiliar items, suggesting that they had abstracted away from particular stimulus items. A follow-up study by Hupbach et al. (2009) found that infants had forgotten specific stimulus sentences 24 h after exposure to the grammar. The abstract information, on the other hand, was retained, but only if a nap had followed shortly after language exposure. Converging evidence from adults comes from a study by Durrant et al. (2011) which showed improved abstraction of statistical patterns underlying tone sequences after a night’s sleep or a brief daytime nap when compared to equivalent periods of wakefulness. The above findings suggest that sleep may contribute to abstraction of statistical regularities, perhaps by promoting a qualitative change in memory which enables greater flexibility in learning (Gómez et al., 2006). Such cognitive flexibility is critical in the process of language acquisition as generalization plays a major role in linguistic productivity.

Statistical learning tasks used to study language learning typically employ artificial miniature languages. The advantage of these languages is that they enable the experimenter to constrain the input in such a way that learning can be attributed solely to the use of those cues directly under experimental control. The main problem with these materials is their low ecological validity. The input participants are exposed to typically lacks the complexity of natural language on a number of dimensions (e.g., acoustic variability, number of words, and frequency of repetition). Thus, it is unclear to what degree findings from the SL literature can be applied to language learning “in the wild.” A goal for future SL studies of language acquisition will be to simulate the complexity of a natural language task while controlling for pre-existing linguistic knowledge as well as the properties of the input. Ideally, such studies should use paradigms where the listener is exposed to auditory and visual stimuli simultaneously to mimic naturalistic learning conditions. Studies incorporating these qualities are beginning to emerge (Gullberg et al., 2010; Hay et al., 2011; Lew-Williams et al., 2011), but at present we need to base our hypotheses about the relationship between language acquisition and SL on studies that have used artificial miniature languages.

While there has been ever increasing interest in SL for more than a decade, it is only in the last few years that researchers have begun focusing on individual differences in this ability and on demonstrating a direct relationship between SL and performance on other cognitive tasks. There is now mounting evidence suggesting that SL is a distinct ability with meaningful individual differences. Kaufman et al. (2010) found variability in SL (using a serial reaction time task) in 153 adolescents aged 16–18 years that was independent of IQ, working memory, and explicit associative learning. The only elementary cognitive task related to SL was processing speed. Arciuli and Simpson (2011) revealed variability in SL in 183 children aged 5–12 years; a finding that is crucial for the argument that SL relates to variability in language *acquisition*.

## Statistical Learning and Language: A Common Neural Basis

There is a growing body of evidence showing that SL recruits the same brain areas as those used in language processing (de Vries et al., 2011; Folia et al., 2011; Petersson et al., 2012). A number of studies using functional magnetic resonance imaging (fMRI) have found that Broca’s area, which is one of the classic language areas, is involved in artificial grammar learning paradigms as well as in the implicit learning of structured motor sequences (Lieberman et al., 2004; Forkstam et al., 2006; Clerget et al., 2012). Corroborating evidence comes from a study using diffusion tensor magnetic resonance imaging (DTI) which found that white matter integrity around Broca’s area predicted performance in an artificial grammar learning task (Floeel et al., 2009). Furthermore, a recent ERP study demonstrated similar neural correlates for a sequential learning task and a language task using a within-subject design (Christiansen et al., 2012).

Studies using repetitive transcranial stimulation (rTMS) and transcranial direct current stimulation (tDCS) have taken these findings a step further by demonstrating a causal relationship between activation in Broca’s area and learning of artificial grammars (Uddén et al., 2008; de Vries et al., 2010). The study by de Vries et al. (2010) is of special interest because it focused on the grammar *acquisition* process rather than the subsequent syntactic judgment. In this experiment three groups of subjects participated in an artificial grammar learning task: one group who received anodal tDCS over Broca’s area, one group who received stimulation over an area which has not been implicated in artificial grammar learning, and one group who received sham stimulation. The group who received stimulation in Broca’s area during the *acquisition* of the grammar performed better than the two other groups in the subsequent grammatical classification task. Interestingly, tDCS over Broca’s area did not significantly enhance working memory, ruling out increased working memory capacity during acquisition as the explanation for the group difference. However, the study employed a between-subjects design, and although an effort was made to match the subjects on a number of criteria, pre-existing group differences may have contributed to the observed effect.

Additional evidence supporting a common neural basis for SL and language comes from investigations of patients with agrammatic aphasia. Christiansen et al. (2010) tested seven patients diagnosed with agrammatic aphasia on a visual SL task. In the training phase of the experiment, patients and control participants were exposed to strings of non-linguistic symbols conforming to an artificial grammar. Both patients and controls performed well in the cover task which involved judging whether one grammatical string matched the next. However, in the test phase where subjects were asked to classify novel strings as either grammatical or ungrammatical, only control participants performed better than chance. Differences between patients and controls could not be attributed to poor visual-perceptual skills or low visuo-spatial working memory in the agrammatic patients. Thus, the results suggest that the language impairment in agrammatic aphasia is associated with impairment in non-linguistic sequence learning, indicating that domain-general neural mechanisms underlie both language and SL. Converging evidence comes from a study by Patel et al. (2008) showing that Broca’s aphasics display impaired processing of structural relations in musical sequences.

Based on this type of evidence, Uddén and Bahlmann (2012) introduced the structured sequence processing perspective which proposes that there are domain general mechanisms in the brain which are common to the processing of structured sequences in language, music, and action. They reviewed a large number of studies which have consistently shown that the left inferior frontal gyrus is engaged in processing of structured sequences independently of whether these are linguistic, musical, or action-related.

## The Association between SL and Proficiency with Spoken Language

There is growing behavioral evidence of an association between SL and language proficiency. Conway et al. (2010) examined the relationship between SL and word predictability in sentence processing in adults. Experiment 1 revealed a positive relationship between visual SL (sequences of colored squares) and auditory sentence processing. Experiment 2 showed a positive relationship between auditory SL (sequences of syllables embedded in pseudospeech) and audiovisual sentence processing. Experiment 3 demonstrated that this relationship was not mediated by immediate verbal recall (digit span) or non-verbal intelligence (Raven’s Progressive Matrices). See Misyak and Christiansen (2012) for an investigation of the link between SL and comprehension of natural language sentences in adults that reported a similar outcome: a relationship between SL and language proficiency that exists independently of cognitive motivation, short-term memory, and fluid intelligence. The findings from these two studies suggest that SL is tapping a distinct capacity.

Consistent with these findings, several studies of language impaired adults have shown poor SL, and that generalization of SL to novel cases appears to represent a particular problem for this population (Plante et al., 2002; Grunow et al., 2006; Richardson et al., 2006; Torkildsen et al., in press). In the study by Grunow et al. (2006) adult subjects with and without language-based learning disabilities listened to strings of three non-words where the first and third word had a dependent relationship. Adults without language impairment were able to learn the non-adjacent contingencies and generalize the underlying structure when variability of the middle element was high (24 unique words), but not when it was low (12 unique words). Adults with language impairment did not show any discrimination between grammatical and ungrammatical strings in either variability condition. Torkildsen et al. (in press) examined the effect of exemplar variability on SL in a simpler learning task, involving adjacent dependencies. Half the learners were exposed to three exemplars of each of the open class elements presented 16 times each (low variability condition), while the other half were exposed 24 exemplars twice (high variability condition). Learners with normal language were able to recognize trained items and generalize the grammar to novel non-word strings in both high and low variability conditions, but relative effect sizes suggested that high variability facilitated learning. In the language impaired group, only those exposed to the high variability condition were able to demonstrate generalization of the grammar. Such evidence has led to the proposal that language impairment may result from a general problem in SL (Hsu and Bishop, 2010; but see Dąbrowska, 2010). However, many studies of adults with language impairment have only examined SL in the verbal domain, making it difficult to disentangle the effects of language impairment and a possible impairment in non-verbal SL.

Examination of the link between individual differences in SL and natural language proficiency is clearly a promising endeavor; however, none of the above studies examined children. To date, only a few studies of children and adolescents have examined the relationship between language proficiency and SL. Tomblin et al. (2007) found that grammar impairments in adolescents were directly associated with low performance on a visual sequential pattern learning task. A recent study by Conway et al. (2011) found that visual sequence learning was significantly correlated with language outcomes in deaf children with cochlear implants. The observed correlations between sequence learning and language were especially robust for a language test measuring the ability to formulate semantically and grammatically correct spoken sentences of increasing length and complexity. The correlation between language and sequence learning was not mediated by either working memory or vocabulary knowledge.

A study by Evans et al. (2009) revealed a link between auditory sequential SL and language proficiency in children aged 6–14 years. They used two tests of SL: (i) syllables in pseudospeech and (ii) sequences of musical tones. Children with SLI performed more poorly than controls on both SL tasks. Children with language impairment did show SL, but required longer exposure to stimuli to learn embedded regularities. After controlling for age, SL during the short exposure condition correlated positively with receptive and expressive vocabulary in typically developing children. After controlling for age, SL during the long exposure condition was positively correlated with receptive vocabulary in children with language impairment. SL was not correlated with IQ in either group of children. In line with Conway et al., this finding suggests that SL is tapping a type of learning that is not assessed by tests of IQ.

As far as we are aware, the only study examining the relationship between an independent test of SL and syntactic acquisition in typically developing children is that reported by Kidd (2012). In this study, 4–6-year-olds were given tests of explicit word pair learning and implicit visual sequence learning in addition to a syntactic priming task. The syntactic priming task included a test phase where children described pictures after they had been primed with a particular syntactic construction (the passive form) and a post-test phase where children described pictures without having been primed. The post-test phase investigated whether priming effects persevered after priming had ceased. Results showed that performance on the implicit SL task predicted maintenance of the syntactic priming effect into the post-test phase of testing. Scores on the explicit learning task, on the other hand, did not predict priming effects. These findings indicate that children’s SL abilities are recruited when learning grammatical usage patterns in input.

The findings reported by Kidd (2012) are consistent with comparable studies of adults such as Conway et al. (2010) and Misyak and Christiansen (2012). However, while the passive form is not typically used by 4–6-years-olds, it is likely that participants in Kidd’s experiment came to the experiment with at least some experience with this construction. Thus, an investigation of an entirely novel syntactic construction would be needed to make claims about the role SL plays in children’s ability to break into the syntactic system that governs their language.

A natural next step to follow up Kidd’s finding is to investigate *how* children make use of the output of SL in the language acquisition process. A recent line of research has set out to examine exactly this question (Graf Estes et al., 2007; Lany and Saffran, 2010, 2011). For example, Graf Estes et al. (2007) asked whether SL during word segmentation yields output that can act as word candidates which can be used in subsequent lexical-semantic acquisition. In the first part of an experiment, 17-month-olds were familiarized with an artificial language where transitional probabilities allowed the segmentation of four words. Next, the infants were taught two novel label-object associations where the labels were either words in the artificial language, sequences that crossed word boundaries in the artificial language (part-words), or words that did not appear in the familiarized language at all (non-words). Graf Estes and colleagues found that infants who had been taught labels that were words in the familiarized speech stream were able to learn the label-object pairings, but infants who were taught part-words or non-words did not demonstrate any learning of the pairings. This result suggests that the output of the SL process can function as input to subsequent word learning.

Mirman et al. (2008) extended this finding by showing that the relationship between statistical segmentation and word learning is also present in adults. However, the authors found a difference between infants and adults in the dynamics between statistical segmentation and word learning. In contrast to infants, who could not learn label-object mappings for part-words or non-words they had not been familiarized with, adults learned words in all three conditions, but were faster in acquiring non-words and familiarized words than part-words. This latter finding suggests that for adults SL has an inhibitory role in hindering the learning of novel meanings for labels that violate learned transitional probabilities (part-words), while for infants SL has a facilitative role in assisting the mapping of labels to novel meanings when labels are consistent with learned transitional probabilities.

Evidence pointing in this direction is not restricted to the area of word learning. A recent study of the acquisition of morphosyntax shows that the non-adjacent dependencies which have the most advantageous distributional patterns are the ones that infants first show evidence of knowing when tested with headturn preference procedures (van Heugten and Johnson, 2010). Thus, there is reason to believe that the output from SL mechanisms is used at various levels of linguistic analysis both by infants and children.

Second-language acquisition (L2) learning is different from first-language (L1) learning in a number of critical ways. Still, it is possible that the detection of statistical regularities plays a role in L2 acquisition. Ellis (2002) argued that both L1 and L2 learning is related to input frequency and its detection and argued that while frequency has been all but ignored in applied linguistics for the last 40 years it may be appropriate to revisit it as a causal factor. Interestingly, a recent study of 153 adolescents demonstrated a significant positive relationship between implicit SL and second language learning of French and German (Kaufman et al., 2010). We do not know of any research that has examined SL in infants living in bilingual environments or any studies that have examined a link between a capacity for SL and proficiency of L2 acquisition; although, it would seem worthwhile to pursue these avenues in future research.

## SL in the Context of Written Language

Both reading and spelling involve learning the correspondences between arbitrary visual symbols and the linguistically meaningful sounds of a language. In English the mapping between letters and sounds can be thought of as probabilistic (e.g., Harm and Seidenberg, 2004; Treiman and Kessler, 2006; Deacon et al., 2008; Kessler, 2009; Seva et al., 2009). For example, the letter “c” often maps onto the phoneme/k/. Of course, “c” can be linked with other phonemes (as in “circle” or “cello”). In the absence of explicit instruction, over time, children are likely to detect contextual cues such as many words beginning with the letter “c” followed directly by the letter “i” have/s/as their initial phoneme. The statistical regularities in written language include non-adjacent pairings (such as “a” later followed by “e”: “cape” versus “cap”). Children are taught explicitly about some of these mappings (and rightly so). Clearly, they are not taught about every single correspondence and contextual cue in English. Surely, that would be impossible.

Arciuli has examined probabilistic cues to lexical stress contained within orthography. For example, corpus analyses have revealed that around 70% of disyllabic English words ending with the letters “-ure” have first syllable stress, whereas around 80% of words ending with “-uct” have second syllable stress. Adults are sensitive to these probabilities. They tend to assign first syllable stress when reading a non-word such as “lenture,” but second syllable stress when reading “feduct” (see Arciuli and Cupples, 2006, regarding cues in word endings and Arciuli and Cupples, 2007, regarding cues in beginnings). A triangulation of (1) corpus analyses of children’s age-appropriate reading materials, (2) behavioral testing across a range of ages, and (3) computational modeling demonstrated that sensitivity to probabilistic cues to lexical stress during reading aloud follows a developmental trajectory in children across the age range of 5–12 years (Arciuli et al., 2010). As children’s exposure to written language increases, sensitivity to these probabilities increases. This sensitivity occurs without having to draw children’s attention to the probabilities explicitly.

The computational modeling component of the study by Arciuli et al. (2010) drew on a single-route connectionist approach to reading in order to explore how children learn to assign lexical stress. Connectionist models operate on the statistical regularities present in the input to which they are exposed. In these models learning occurs via adjustment of the weights on connections between units in order to approximate a target response. Gradually, these connection weights are altered in order to increase the accuracy of the model’s response. Importantly, connectionist models can be trained iteratively enabling us to explore developmental trajectories based on age-appropriate input. Thus, connectionist models embody the principle of SL. For many years cognitive scientists have contrasted connectionist approaches where a system learns regularities with an alternative approach where pre-determined rules are utilized. For example, Rastle and Coltheart (2000) reported on a rule-based algorithm for stress assignment as part of the dual-route cascaded model of reading that was designed to simulate the reading aloud of disyllabic non-words. The algorithm involved searching through the letter string of a non-word for morphemes (to identify a specified set of affixes: 54 prefixes and 101 suffixes), and then consulted a database for information concerning whether each morpheme carried stress or not (e.g., the suffix“-ing” does not carry lexical stress). The algorithm successfully simulated some aspects of stress assignment in adults’ reading; however, it was difficult to see how children might come to acquire such a system. How might children learn what constitutes a prefix and what constitutes a suffix? How might children end up with a store of knowledge pertaining to whether affixes carry lexical stress or not? More recent instantiations of the dual-route model of the reading aloud of polysyllables have incorporated connectionist principles (e.g., Perry et al., 2010).

The debate about rules versus statistics and whether some kind of hybrid system might best for explaining language acquisition continues (Newport, 2010). Connectionist modeling has a central role in this debate. For example, connectionist modeling has been used by researchers interested in the so-called “more than one mechanism” (MOM) hypothesis of language acquisition. According to the MOM hypothesis language is acquired via both rule-based and statistical mechanisms. Some researchers have used under performance of a connectionist model in simulating human data as evidence in favor of MOM (Endress and Bonatti, 2007) while others have used connectionist modeling to directly rebuke such claims (Laakso and Calvo, 2011).

Arciuli and Paul (2012) examined sensitivity to probabilistic orthographic cues to lexical stress in adolescents with autism compared with matched typically developing peers (all participants were 13–17 years; groups were matched on age, verbal IQ, spoken language, and reading ability). Using the stimuli and silent reading task from Arciuli and Cupples (2006) they demonstrated that adolescents with autism lack sensitivity to these cues. There was no requirement to produce individual words, so it seems unlikely that motor explanations can account for this finding. They discuss the possibility that some individuals with autism lack the ability to “tune in” to the details of ambient language (Shriberg et al., 2011). Arciuli and Paul suggested that this lack of attunement may be related more generally to impaired SL. An fMRI study by Scott-van Zeeland et al. (2010) revealed a lack of SL during exposure to artificial language containing statistical regularities in individuals with ASD (9–16 years). In contrast, behavioral research has indicated that implicit learning is intact in individuals (8–14 years) with autism (Brown et al., 2010). More research is needed to clarify whether SL is impaired in autism. In keeping with what we know about variability of SL in typically developing individuals (e.g., Arciuli and Simpson, 2011), it seems likely that there is also variability in SL ability in the autism population. This may explain why some group studies find impaired SL in autism while others do not. It is worth noting the suggestion that social cues may enhance children’s implicit learning by highlighting *what* it is that is to be learned and *when* it ought to be learned (Meltzoff et al., 2009). It may be that some children with autism are not sensitive to the kinds of social cues that support SL (see also Tomasello, 2010).

Arciuli and Simpson (2012b) examined the relationship between SL and reading aloud in typically developing children and healthy adults. SL was assessed using sequences of visually presented items, a variation of the triplet-learning paradigm. Reading accuracy was assessed using a standardized test of single word reading. This constituted a highly conservative test of the hypothesis that an individual’s capacity for SL might be related to their reading proficiency: the SL task used non-linguistic stimuli bearing no particular resemblance to the reading process, while the reading task had not been designed with an emphasis on the probabilistic relationship between letters and sounds. The data revealed a significant positive relationship between SL and reading proficiency in children and also in adults, even after age and attention were taken into consideration. Neither phonological working memory nor non-verbal IQ mediated the relationship between SL and reading ability.

Presumably, a capacity for SL could facilitate the acquisition of written language directly (there are many statistical regularities in written language) as well as indirectly via links with oral language proficiency (it is well known that reading and spelling ability is closely related to oral language ability). We are not aware of any research that has examined whether infants’ capacity for SL is related to their proficiency with written language in later years.

## The Potential of Longitudinal Research

Solid progress has been made in supplying the kind of empirical evidence required to demonstrate that SL plays a role in language acquisition. Especially helpful in this regard are recent studies that have shown a link between performance on a test of SL and performance on a test of language proficiency, as well as studies demonstrating how infants and adults use the output of the SL process in subsequent lexical acquisition. We have now reached a point where longitudinal research is needed to assist in furthering the language acquisition debate. Longitudinal studies cannot prove causality, but they are a vital step in exploring the nature of a relationship once an association between variables has been discovered, and a necessary step before intervention studies targeted at those with impairments can be considered.

While we know of no previous studies which have investigated a direct link between SL and later language outcomes, there are longitudinal studies showing that speech segmentation, phonological discrimination, and non-linguistic auditory processing abilities during the first year of life, abilities which may be associated with SL, predict later language outcomes (Newman et al., 2006; Kuhl et al., 2008; Choudhury and Benasich, 2011). There are also longitudinal studies of toddlers in their second or third years demonstrating that lexical processing skills in meaningful contexts predict later language outcomes. Marchman and Fernald (2008) found that speed of spoken word recognition and vocabulary size at 25 months predicted language skills at 8 years of age. In a more recent study, Fernald and Marchman (2012) extended these findings by showing that word recognition at an even younger age, 18 months, predicted vocabulary growth into the second half of the third year in typically developing and late talking toddlers.

These findings demonstrate that longitudinal research beginning in the first or second year has great potential for investigating the influence of various cognitive abilities on language development. However, such longitudinal studies present a number of challenges. One of these is that there is great variability in infants’ ability to successfully complete behavioral and electrophysiological assessments at this age. Since longitudinal studies are costly and time-consuming, many researchers are forced to keep the data collection period as short as possible, over only a year or two.

Moreover, some very early language assessments (at age 18–24 months) have shown poor sensitivity and specificity in predicting language outcomes only a year later. Some 2-year-olds turn out to be “late bloomers,” and a fair proportion of others who had age-appropriate language at age 2 meet the criteria for language delay at age 3 (Dale et al., 2003; Henrichs et al., 2011). One option is to begin testing a little later, around 3 years of age, and follow up with subsequent testing of oral and written language proficiency thereafter.

Certainly, longitudinal research will need to investigate SL in relation to acquisition in different linguistic domains (e.g., vocabulary and morphosyntax; oral versus written language) and strive to employ more naturalistic stimulus materials than those which have traditionally been used. Ideally, behavioral studies tapping SL and linguistic knowledge at developmentally significant ages need to be combined with corpus analyses to obtain a realistic picture of the input that children receive. This kind of research can be used in conjunction with computational models and neuroimaging to explore possible mechanisms that give rise to developmental changes in behavior.

Longitudinal investigation of whether early SL ability is related to later language proficiency is an important step toward the design of intervention studies which in turn can be used to examine causality. For example, in the area of SLI it has been explicitly stated that “The extent to which deficits in statistical learning could supplement extant theories, such as deficits in working memory, in the literature of SLI requires further empirical examination… this line of research can potentially provide useful information for future development of intervention programs” (Hsu and Bishop, 2010, p. 275). In terms of treatment possibilities, increasing participants’ exposure to particular linguistic constructions (such as those in some relative clauses) can make them easier to learn (Wells et al., 2009). Another line of research with clinical relevance are studies that have demonstrated the benefit of high variability for learning morpho-syntactic relations (Gómez, 2002; Gómez and Maye, 2005; Torkildsen et al., in press). These studies indicate that the structure of the learning context can determine whether a particular grammar is learned and generalized. This is an especially relevant finding, given that failure to generalize learning has been identified as a significant problem for those with impaired language. Thus, language impairments associated with inefficient SL might potentially be remediated by focusing on the salience, volume, and/or variability of the input provided to learners. Assessment of SL may also assist early identification of risk/impairment so that other evidence-based interventions can be introduced.

In sum, it has been well established that many infants, children, adolescents, and adults are equipped with highly efficient abilities to detect statistical regularities in input. Recent research has brought the knowledge that humans use the output from these statistical mechanisms in language acquisition and that individual differences in SL are related to language proficiency. Longitudinal studies are needed to determine the extent to which SL contributes to the transition from non-linguistic infant to fully fledged language user in typically developing individuals and the extent to which impaired SL presents challenges for those with disorders such as autism, SLI, and dyslexia.

## Conflict of Interest Statement

The authors declare that the research was conducted in the absence of any commercial or financial relationships that could be construed as a potential conflict of interest.
